# Conditions and Dynamics That Impact Maternal Health Literacy among High Risk Prenatal-Interconceptional Women

**DOI:** 10.3390/ijerph15071383

**Published:** 2018-07-02

**Authors:** Suzanne D. Thomas, Sandra C. Mobley, Jodi L. Hudgins, Donald E. Sutherland, Sandra B. Inglett, Brittany L. Ange

**Affiliations:** 1 CSRA Nursing Associates, PC, 300 Gardners Mill Court, Augusta, GA 30907, USA; 2 Department of Obstetrics & Gynecology, Medical College of Georgia, Augusta University, Augusta, GA 30912, USA; samobley@augusta.edu; 3 Education and Networking, Enterprise Community Healthy Start, Augusta University, Augusta, GA 30912, USA; jhudgins@augusta.edu; 4 Enterprise Community Healthy Start, The Perinatal Center, Augusta University, Augusta, GA 30912, USA; dsutherl@augusta.edu; 5 Enterprise Community Healthy Start, College of Nursing, Augusta University, EC-5338, 987 St. Sebastian Way, Augusta, GA 30912, USA; singlett@augusta.edu; 6 Department of Population Health, Division of Biostatistics and Data Science, Augusta University, Augusta, GA 30912, USA; bange@augusta.edu

**Keywords:** determinants of health, health disparities, health education, health literacy, health interventions, health promotion, social disadvantage

## Abstract

The purpose of the study was to describe conditions and dynamics in the lives of high-risk, low-income, Southern United States prenatal-interconceptional women (*n* = 37) in a home visiting program that promoted maternal health literacy progression. In the Life Course Health Development (LCHD) Model, conditions were risk and protective factors that impacted health. Dynamics drove the complex, epigenetic relationships between risk and protective factors. Maternal health literacy promotion helped participants address conditions and dynamics to create positive life changes. This research was a retrospective, mixed methods study of women’s service records documenting care from prenatal admission to 24 months post-delivery. The Life Skills Progression Instrument (LSP) was scored to measure maternal health literacy progression. Ethnographic content analysis of visit notes triangulated with quantitative data enabled specificity of critical data elements. Subsequently, a complementary focus group was conducted with the Registered Nurse Case Managers (RNCM). Severe social conditions included devastating poverty, low educational achievement, transient housing, unstable relationships, incarceration, lack of continuous health insurance, and shortage of health care providers. Dynamics included severe psycho-social stressors, domestic violence, lack of employment, low income, low self-esteem and self-expectations, and social/family restraints upon women’s intended positive changes. An important protective factor was the consistent, stable, evidence-informed relationship with the RNCM. Findings from the focus group discussion supported content analysis results.

## 1. Introduction—Purpose and Theoretical Framework

The purpose of this study was to describe conditions and dynamics in the lives of high-risk, low-income, Southern United States prenatal-interconceptional women, who were enrolled in a U.S. government-funded home visiting program called Healthy Start. Healthy Start was originally charged with reducing infant mortality. In time, it became clear that medical care alone was not able to accomplish that goal, and that women and their infants needed a much broader approach to care. The Enterprise Community Healthy Start (ECHS) program chose to promote functional health literacy among the prenatal-interconceptional women who participated in the ECHS program. Prenatal meant from pregnancy conception to delivery. Interconceptional meant after delivery through 24 months of the infant’s life. Increased functional health literacy among prenatal-interconceptional women was termed maternal health literacy progression.

Women or participants were considered to have high-risk conditions if their medical, social, environmental, physical, interpersonal, financial, educational, or any other socio-economic factors were judged as hazardous to the well-being of the woman or her fetus or infant. Thus, conditions were risk factors. Conditions also included factors that were protective or helpful in reducing risks to the woman and her fetus or infant. Dynamics described how risk and protective factors interacted. In theory, dynamics among risk and protective factors were powerful enough to limit or increase gene expression. Examples of dynamics included social or peer pressure, financial incentives or costs, physical or physiologic changes associated with pregnancy, and the search for safe housing, for love and belonging, and for reproduction. The Life Course Health Development Model (LCHD) described epigenetic interaction, which meant the ways that conditions and dynamics worked together to bring about changes in health development over the course of a person’s life [1,2,3]. The programmatic goal of ECHS was to intervene in participants’ lives to reduce their risks and bring about positive health developmental potentials for participants and their infants.

### 1.1. Maternal Health Literacy Progression

Functional health literacy was defined as a person’s knowledge, skill, and ability to understand and apply health related information and to access health care services [4]. The recent return to home visitation as a model of service delivery showed promise to examine the unique setting where health related decisions and activities took place, to understand women’s conditions and dynamics, and to strategize actions that promoted health [5,6]. Investigators conceptualized these health promotion strategies as two theoretical constructs in maternal health literacy [4,7,8,9]. First, Maternal Health Care Literacy was conceptualized as a participant’s knowledge, skill, and access to health care, and her ability to manage her own and her infant’s care; and second, Maternal Self Care Literacy was conceptualized as her self-health practices, self-esteem, use of resources, and knowledge of infant development. The home environment’s uniqueness made it difficult to measure the impact of nursing services upon maternal health literacy. Thus, in 2005, ECHS adopted the Life Skills Progression Instrument (LSP) as a structure for care and as a means of recording observed changes in maternal health literacy among women who were participants in ECHS services [10]. Progression in maternal health literacy was understood to indicate participants’ increasing health equity, so that participants’ health outcomes were closer to those of higher income, better educated, low-risk women’s health outcomes [1,11]. ECHS promoted maternal health literacy progression through health education and health counseling, referrals to community and medical resources, and support through a consistent long-term relationship between a participant and a Registered Nurse Case Manager (RNCM).

An initial study documented that greater than ninety percent of participants in the ECHS Program made positive maternal health literacy progression from their prenatal to their postpartum assessments. Following delivery through 24 months of the infant’s life, participants faced new challenges, and approximately 35% (*n* = 37) did not meet the measured criterion for adequacy in maternal health literacy progression at their final assessment. However, these participants did meet the most critical measure of all: they and their infants survived pregnancy, birth, and the first two years of their infants’ lives [4]. In this report investigators examined the conditions and dynamics in the lives of participants who did not reach adequacy in maternal health literacy progression, and how their Registered Nurses who served as case managers intervened to improve their long-term health development potentials.

### 1.2. Setting and Background

The ECHS service area consisted of two non-contiguous rural counties in east central Georgia, U.S. These two counties ranked among the poorest in health status and health outcomes in the state (156th and 144th of 157 counties ranked), and in the nation (37th of 50 states) [12]. One service county had a community hospital that provided delivery services, and there was a board-certified obstetrics physician who practiced in that county. All other deliveries were made in the regional medical center that was an average of 25 miles away for ECHS participants. There was no public transportation in the two counties. Little or no primary medical care for the underinsured and uninsured was available in the two service counties. There were health professional shortages in primary medical, dental, and mental health services [13].

Rates of U.S. teen pregnancy, abortion, and infant mortality fell during the data collection period of the study, 2005–2012. When infants were born preterm, at less than 37 weeks of gestation, they were much more likely to die before their first year of life. Preterm birth was the risk most closely associated with infant mortality and had its highest rates among non-Hispanic black NHB infants. Compared to the 2014 national rate of 9.57% [14,15], the preterm birth rate in the Southern state of Georgia was 10.8%, and, in the Augusta, GA Perinatal Region that encompassed the study population, the rate was 12.6%.

Interpregnancy interval was the time from delivery to the next pregnancy conception. Short interpregnancy intervals of less than nine months, and very short interpregnancy intervals of less than three months, were associated with both preterm birth and morbidity for women and infants. Teen pregnancy was an important risk factor for women and infants because most teens were not married, they had not completed their education, and they were not mature physically or emotionally. Although it was a goal of the U.S. Department of Health and Human Services (USDHHS) to reduce the rates of teen pregnancy, teen pregnancy rates were still very high in the U.S. and Georgia during the study period. Teens were the most likely age group to have short interpregnancy intervals [16,17].

Even though teen pregnancy, abortion, and infant mortality rates declined for all three ethnic and racial groups, those for NHB women and infants did not decline as rapidly. Thus, disparities widened among NHB women and infants and other ethnic and racial groups [18]. NHB women in Georgia suffered a preterm birth rate three times greater than that of non-Hispanic white (NHW) women. Over the years, the difference in the rate of premature births widened as the NHW prematurity rate declined faster than the NHB rate [15,19]. Thus, racial disparities grew in the U.S. and in Georgia among women and infants of color (NHB) compared to NHW women and to women of all other races or ethnic groups.

Another important goal of the U.S. DHHS was to prevent pregnancy-related death (MMR), which was the number of maternal deaths within one year of pregnancy per 100,000 live births. It was recorded as the maternal mortality ratio (MMR). In 2013, Georgia’s MMR was 24.9 compared to the U.S.’s ratio of 17.3 [19,20]. The U.S. Centers for Disease Control and Prevention (CDC) reported wide racial disparities in MMR in the years 2011–2013. NHB women died at the rate of 43.5, while NHW women died at the rate of 12.7, and women of all other races and ethnic groups combined died at the rate of 14.4 [21]. These data demonstrated the great differences in MMR that occurred when NHB women were compared to women of other ethnic and racial groups.

The U.S. CDC routinely conducted surveys of women who delivered a live infant. The survey was called PRAMS or Pregnancy Risk Assessment and Monitoring System. Based on earlier studies from the PRAMS data, investigators knew that social and demographic risk factors for maternal or infant mortality rates included the following: low income, NHB race, poor mental health, interpersonal violence, and substance abuse [21].

Stress was a high risk factor associated with depression, hypertension, and alcohol, tobacco, and drug use. Severe stress during or after pregnancy occurred if a parent—mother or father of the baby—was incarcerated [22]. Among very poor people with little education, and few chances for employment, there were very few marriages. Without marriage, family relationships were uncertain and unstable. When a pregnant woman was not married, the absence of the baby’s father contributed to the risks of maternal and infant morbidity [23,24].

Healthy Start programs, including the ECHS program, were charged with discovering what the problems were that led to so many women and infants dying and intervening to prevent their deaths. NHB women and their infants were a special focus since their rates were so much higher than others’ rates. Investigators chose to examine this group of ECHS participants because they were at greatest risk of adverse outcomes.

## 2. Materials and Methods

### 2.1. Ethics

This research was conducted in accord with prevailing ethical principles and was approved by the Georgia Regents University (now Augusta University) Institutional Review Board. Original empirical research was conducted under Research Protocol #1010080 for evaluation of participant service and under Research Protocol #716980 for the RNCMs to participate as subjects in a focus group. These protocols were approved by the Institutional Review Board. Throughout the study, investigators protected participants’ personal health information. Each investigator was required to demonstrate knowledge of and practice in the highest standards of ethical behavior in research prior to their participation in the study.

### 2.2. Design

The study was a mixed methods design with ethnographic retrospective content analysis of prenatal-interconceptional service records for 37 ECHS participants [25]. Using techniques appropriate for mixed methods design specified by Sandelowski [26], investigators: (1) triangulated content analysis data with multiple sources of quantitative data to specify critical data elements, (2) complemented content analysis with a focus group comprised of the RNCMs to further explicate findings from content analysis, and (3) upon the recommendation of the RNCMs, planned additional studies to follow up 23 of the 37 study group participants who returned to ECHS to have nursing services for their next pregnancy.

### 2.3. Study Participant Recruitment and Description

Participants were referred to ECHS by local and regional providers because of their high-risk prenatal status. Participants lived in two rural southeastern Georgia counties with limited community resources, and health professional shortages in primary medical, dental, and mental health services [27]. Services were provided during 2005–2012 from prenatal entry into case management and up to 24 months post-delivery. Study participants were twenty-eight (78.8%) NHB and nine (24.3%) NHWs. Entry into the study was determined by their inability to achieve the criterion for adequacy in maternal health literacy progression as evidenced by their final comprehensive postpartum numeric scores on the LSP instrument [10]. Education was an important indicator of a woman’s readiness to learn parenting and self-care skills. Most (*n* = 21, 57%) participants had less than a twelfth-grade education; their ages ranged from 14 to 36 years (median = 18.5, mode = 19). Eleven of the 37 (29.7%) were less than 19 years of age when admitted to prenatal care. One teen was gravida 2; 10 teens, gravida 1. Mean gravidity was 1.8 pregnancies (s d ± 1.4). Mean gestational age at delivery was 38.6 weeks (s d ± 2.2); two (5.4%) delivered at <37 weeks gestation. Mean birthweight was 3095.6 g (s d ± 551.7), and four (10.8%) infants had <2500 g low or very low birthweights (range: 1360–2381 g).

Risk factors from initial prenatal screening included: severe social conditions (12), late or no prenatal care (8), standard body weight >20% (8), obesity (3), positive depression screen (6), depression (4), severe mental disorder (2), hypertension (4), tobacco dependence (3), gestational diabetes (3), last delivery <1 year ago (4), history of preterm labor (3), parent of a NICU (neonatal intensive care unit) graduate (2), previous preterm birth (2), previous cesarean birth (2), under age 15 at conception (2), and family history of breast cancer (2). Thirty-four women had 2 or more risks (median = 4, range = 1–8 risks).

### 2.4. Intervention

The ECHS intervention program promoted maternal health literacy by teaching participants to manage their own and their infant’s health care, including self-care practices, use of resources, and promotion of infant development. RNCMs used and taught participants a think-link-respond approach to problem solving, thus teaching them to consider consequences and make deliberate choices instead of reacting to circumstances. This critical thinking skill gave participants the opportunity to make life changes.

The four RNCMs were culturally and linguistically similar to participants. They provided nursing services to consistently assigned participants, using collaborative, interactive one-on-one visits with participants in their homes, schools, community settings, and in RNCMs’ private offices. RNCMs facilitated access to the health care system, served as advocates, made referrals, and tracked follow-through of referrals. RNCMs maintained communication with participants using cell phones to text and talk as needed.

RNCMs conducted ongoing health counseling and education. Content was structured around client-identified needs using the Beginnings Guides for Pregnancy and Parenting [28]. RNCMs stimulated problem solving skills using reflective function [29]. They monitored physical, psycho-social, and environmental status. Participants had home visitation nursing services for a range of 10.2–29.7 months (median = 20.7 months). RNCMs increased frequency and intensity of visits as indicated.

Based on Kotelchuck’s Adequacy of Prenatal Care Utilization Index, thirty-one participants had adequate to adequate-plus medical prenatal care as indicated for very high-risk pregnancies; eight had late entry into prenatal care [30]. Referrals for maternal-fetal medical care or other specialized health care involved arranging private or Medicaid transportation to the regional perinatal center. Children were not allowed to ride on the Medicaid bus unless it was for their pediatric appointment. Participants had limited access to child care and were reluctant to be away from home when older children came home from school.

Insurance was critical for access to medical care in the United States. During the preconceptional period twenty participants had no health insurance and no access to medical care even though they had very high medical risks. The lack of health insurance before and after pregnancy accentuated their health risks. The untreated high risk medical problems observed in this young study group of women was reflective of the lack of health insurance and health services available in their communities. RNCMs assisted all participants to access health insurance and health care during and after pregnancy through advocacy, guidance, and persistent support.

### 2.5. Instrumentation

The LSP was a validated set of items that measured factors important for home visitation with mothers, infants, and families of young children (alpha range 0.64–0.96) [10]. Groups of items were not considered scales. Items were scored with a rubric on a relational, numeric scale (0–5). Individual items as well as the groups of items representing the constructs Maternal Health Care Literacy and Maternal Self Care Literacy were scored as “adequate” if scores were equal to or greater than four (≥4). Items that were used to represent Maternal Health Care Literacy and Maternal Self Care Literacy demonstrated a consistent meaning that represented the constructs accurately, as evidenced in practice outcomes [4,8]. RNCMs used the LSP to identify women with adequate versus inadequate maternal health literacy progression. LSP items, scoring frequencies, item descriptions and the sample selection process were presented in earlier reports [4,27].

### 2.6. Data Collection

ECHS adopted the LSP on 1 July 2005. Staff had initial and continuous training with the LSP throughout the study period in formal and informal sessions in monthly case conferences. Through the training process RNCMs developed a clinical subculture of shared meanings, procedures, scoring, and likely interventions based upon nursing process that demonstrated internal consistency among RNCMs’ scores for like phenomena.

During the prenatal period, RNCMs observed and interacted with participants over a period of at least two months prior to scoring the prenatal LSP. Within the first two to four weeks after the infant was in the home, an initial postpartum LSP assessment was conducted. Subsequent LSP assessments were conducted every six months, although participants had multiple contacts between LSP assessments. RNCMs recorded all data within 24 h of the dates of contact in the perinatal database, an electronic health record created by and for the ECHS program. Access was password protected and limited to employees and nursing faculty. Provisions were in place for a participant to have a copy of her own data upon request.

RNCMs screened participants with the Beck Depression Inventory [31] and Edinburgh Postpartum Depression Survey [32] upon entry into prenatal care, during the third trimester, and soon after delivery. Both instruments demonstrated validity and reliability with multi-racial, multi-cultural perinatal populations. ECHS collected demographic and identifying data, as well as periodic risk assessments and items about social and financial support. ECHS also obtained information from outpatient medical care, public health visits, schools and social services, and hospital discharge summaries on prenatal, delivery, and interconceptional admissions for the participant and her newborn infant. All data were recorded in the perinatal data base.

### 2.7. Investigators’ Reflexivity

The four Registered Nurse investigators shared personal orientations and motivations before they conducted the research. Rules for group process and privacy were established and consistently followed. Investigators had advanced preparation at the master’s and doctoral level in nursing with research, teaching, clinical, and administrative nursing experience. The database manager was doctorally-level prepared in a health-related field. The statistician had a master’s degree and was enrolled in doctoral studies. Each investigator had a unique contribution that was incorporated in the research process.

Investigators were females, white, middle-later age, middle income and either currently or previously married. RNCMs were females, black, young adult to middle age, middle income. Three were currently or formerly married, and one had never married. Participants were low-income, black (28), white (9), not married (35), married (2), teens (15), and adults (22), and had low educational achievement. RNCMs provided a bridge across the cultural, educational, social, and linguistic differences noted between investigators and participants.

### 2.8. Data Analysis

Content analysis was guided by Berg’s ethnographic method [25]. Investigators worked from a COREQ outline to structure the process of the study [33]. The unit of analysis was the individual participant. Visit notes consisted of the written record of the RNCMs’ activities and observations about participants’ conditions, dynamics, and responses to care. Triangulation incorporated each participant’s data from all sources that were collected and recorded in the perinatal data base prior to the date of each LSP score. Investigators did not critique RNCMs’ work. The database manager transferred quantitative data to an Excel spreadsheet for analysis and printed qualitative data from individual records. Data were checked for accuracy. To maintain confidentiality, files were encrypted and shared among the research team on the university’s secure server. Investigators met in a private conference room or joined meetings on a secure conference line [34].

One investigator analyzed LSP data using Microsoft EXCEL© (Microsoft Corporation, Redmond, WA, USA) to examine the 20 LSP items scored for each assessment for each woman on the two constructs, Maternal Health Care Literacy and Maternal Self Care Literacy. Participants were those who had a final postpartum score less than four (<4) that indicated inadequate progression in Maternal Health Care Literacy and/or Maternal Self Care Literacy. During content analysis sessions, investigators triangulated the scored LSP items at each assessment with the dates of changes in LSP item scores, and the date-related visit notes and other available data for the period preceding the LSP assessment.

Investigators created a form that was used for each participant’s case review. In the first column categories were problems, responses to care, resources, possible intervention to strengthen resources, barriers, possible intervention to remove barriers, interventions attempted and outcomes, and investigator’s professional opinion. In the second column, each section of notes identified RNCM’s factual statements, RNCM’s interpretation of facts, and investigators’ statement of problems. A third column was used to record supporting data and literature references. Investigators reviewed each participant’s data as a group.

During content analysis sessions, one investigator served as recorder using Microsoft Word© (Microsoft Corporation, Redmond, WA, USA), on a Toshiba laptop computer (Toshiba USA Corporate Office Headquarters, Toshiba America, Inc., 1251 Avenue of the Americas, Ste. 4110, New York, NY 10020, USA—manufactured in China 6060B1023401 CM-2). The recorder projected onto a blank wall investigators’ observations, interpretations, and commentary in real time. Following each session, the recording investigator sent encrypted case review forms and notes to each investigator on the university’s secure email server.

### 2.9. Design Complementarity

**Figure 1:**
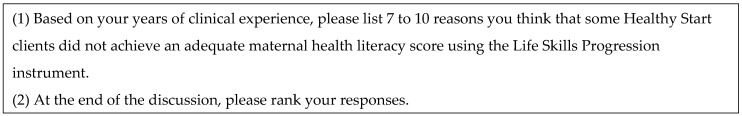
Focus Group Questions. The recorder grouped and recorded RNCMs’ ranked responses with their input.

Following content analysis, investigators invited the RNCMs to participate in a focus group discussion at the end of the study that would serve as an external validity check for data interpretation [26]. Investigators did not contribute to the discussion. RNCMs had study questions in advance. (See Figure 1). Each RNCM had a list of the 37 participants whom they served. Their comments were recorded in their presence as they occurred, projected onto a blank wall, and clarified with their feedback. Responses were not subjected to further analysis. Minimal editing was done to preserve their meaning.

### 2.10. Design Development

Design development is the third stage of the technical levels in mixed methods studies according to Sandelowski [26]. Investigators planned additional studies from existing data for 23 of the 37 study group participants who returned to ECHS for their next pregnancy.

## 3. Results

### 3.1. Content Analysis

#### 3.1.1. Risks

Content analysis revealed multiple risks. Of the 37 participants in the study 35 (95%) had unstable family relationships that were characterized by severe social conditions. Common themes of severe social conditions included: extreme poverty, incarceration of seven fathers of babies (FOBs) and one participant; transient, inadequate housing, and/or crowding; domestic violence; lack of safety; lack of privacy; and no protection from others in the household. Not one of these participants was in charge of the place she stayed. Participants were functionally homeless, staying short periods with their relatives or the FOB’s family, with no permanent home. Patterns of toxic stress were evidenced by anger, grief, depression, fighting, and drug use. Additional stressors were from family members’ criminal activities and extreme multi-generational poverty. Participants and FOBs lacked skills for employment and/or had unstable employment in low-income jobs. 

#### 3.1.2. Protective Factors

The most important protective factor was the consistent, long-term relationship with the RNCM that was focused upon maternal health literacy progression. While pregnant, participants were enrolled in Medicaid and had access to medical care. The RNCMs worked closely with medical providers to monitor medical conditions and teach participants to implement medical recommendations. The RNCMs also worked closely with DFCS social workers, local law enforcement personnel, and other community resource persons to ensure the safety and security of participants and their infants. The RNCMs provided continuous support and guidance through cell phone communications. The RNCMs provided a semi-structured program of maternal health education, infant care, developmental monitoring, and referrals to needed resources as appropriate, by teaching, questioning, showing examples, and by health counseling. As persons with cultural and linguistic similarity who were successful role models, their guidance held the respect of participants and their families. Even so, the RNCMs had limited ability to intervene in participants’ severe social conditions and dynamics.

#### 3.1.3. Dynamics

The competition for owning the infant’s right to an income tax credit was a driving force in the participants’ finances. There was no or limited evidence of any FOBs’ financial support. If the participant added the FOB’s name to the infant’s birth certificate, the FOB became legally liable for financial support, and others in the participant’s household could not receive tax credit for supporting the infant.

Social pressure between participants and their babies’ fathers created unstable, highly stressful family relationships with personal, social, financial, and legal implications. Participants reported that the FOB was “controlling”, “FOB was upset with her”, because she applied for child support, and “off and on relationship with FOB to get his support for their baby”. Therefore, participants did not usually include the FOB’s name on the infant’s birth certificate.

Existing social structures within families limited participants’ abilities to change. Typically, participants had poor responses to referrals for life improvements. Overwhelming barriers and stressors, including family members’ resistance, defeated most efforts to change.

The demands of single parenting focused participants’ resources upon their infants. Infant wellness and sick care, rather than postpartum and interconceptional maternal self-care, were high priority as evidenced by multiple health care contacts per child.

#### 3.1.4. Patterns

**Figure 2:**
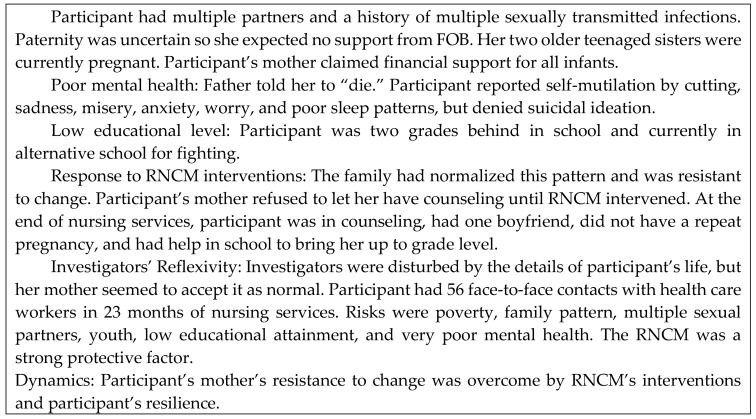
Exemplar: Health Consequences of Teen Pregnancy—A Dysfunctional Family Pattern. Intensive nursing care enabled this family to change to a more health-promoting family pattern.

**Figure 3:**
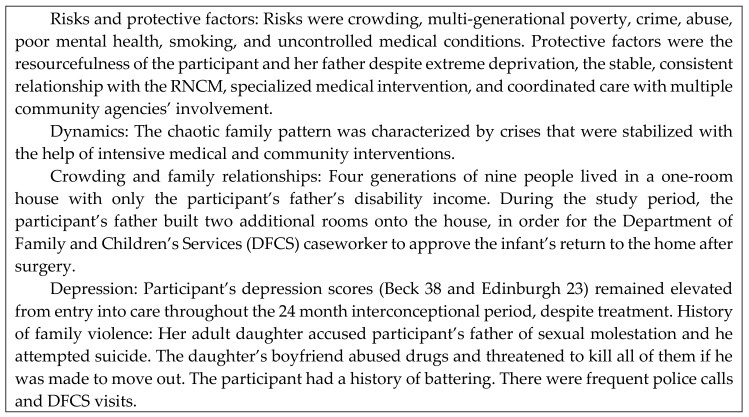

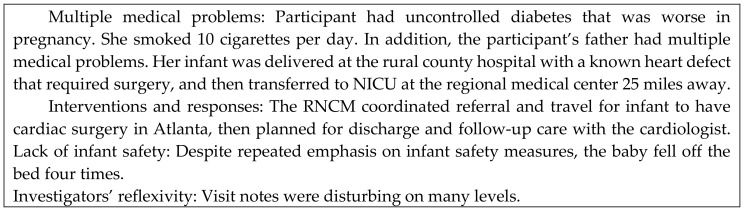
Exemplar: Health Consequences of a Chaotic, Dysfunctional Family Pattern with Multi-Generational Poverty and Family Violence. Intensive nursing care enabled this family to change to a more health-promoting family pattern.

Patterns were regularly occurring sets of conditions and dynamics by which families or household groups conducted their activities of daily living. When one or more family or group member benefitted at the expense of others, the pattern was dysfunctional. When family or group members were committed to the well-being of one another, the pattern was functional. Participants’ development of skills for maternal health literacy was affected by family patterns. Two examples (exemplars) that illustrate how participants’ conditions and dynamics affected their maternal health literacy are shown in Figure 2 and Figure 3. 

Figure 2 contains an example of a family pattern in which the matriarch of the household encouraged her teenage daughters to have multiple pregnancies with multiple FOBs. The matriarch then claimed the infants’ tax credits to control the family finances. The teenaged daughters were unable to complete high school. They were exposed to multiple risks of sexually transmitted infection. And they and their infants were on a pathway to living the rest of their lives in severe poverty as was the participant’s mother. (See Figure 2.)

Figure 3 shows the results of a disorganized family pattern in which individuals strived to meet their own needs at the expense of others, resulting in chaos and poor health. The jeopardy for the newborn infant was illustrated by the participant’s delivery in a local community hospital with no advanced level of treatment when she knew that her infant had been diagnosed in utero (while she was pregnant) with a heart defect. Further jeopardy was seen in the infant’s four times falling off the bed. The RNCM’s timely intervention saved this infant’s life more than once. (See Figure 3.) 

### 3.2. Focus Group Results

#### RNCM’s Focus Group Responses

**Figure 4:**
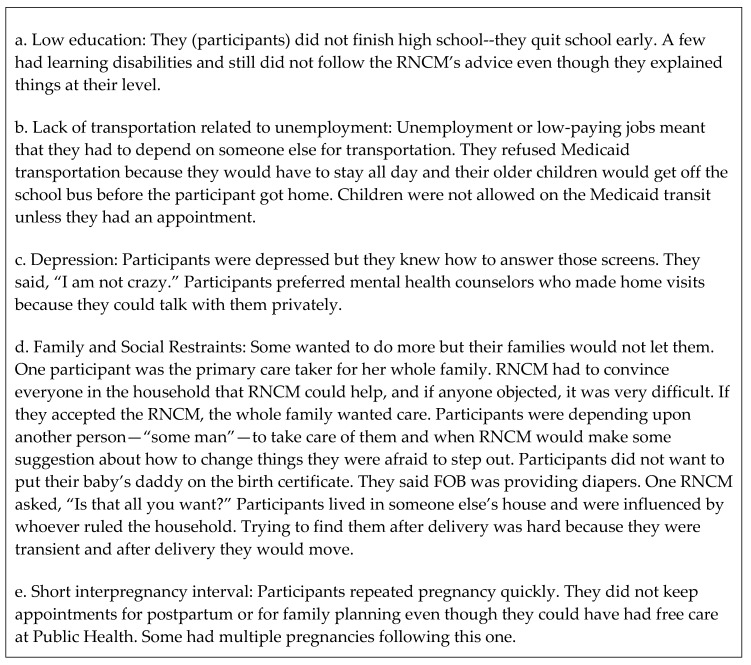
Results of Focus Group with RNCMs. The focus group with the RNCM providers was a complementary research strategy that clarified and extended the content analysis.

Focus Group Results are shown in Figure 4. They were recorded in the RNCMs’ own words as presented here. RNCMs’ recommendations are presented in the final recommendations section of this report. (See Figure 4.) 

The language in Figure 4 was a real-time recording of RNCMs’ comments in their own words; some were colloquial statements. “… wanted to do more …” meant that some participants wanted to change their circumstances. “… afraid to step out …” meant that participants feared the consequences if they took recommended actions to change their circumstances. RNCMs’ recommendations have been placed at the end of this report to emphasize their importance.

### 3.3. Additional Planned Studies and Interventions

Investigators planned to calculate interpregnancy intervals and note the impact of a second session of ECHS intensive home nursing services upon maternal health literacy progression scores. Additional interventions were developed and implemented, including a module on Reproductive Life Planning.

## 4. Discussion

Data illustrated the critical influence of sociodemographic factors upon the health of participants and infants and their inadequate maternal health literacy progression [35]. Severe social conditions affected 35 of 37 participants. Participants lived with devastating poverty. They had little control over the conditions and dynamics of their lives and had continuous struggles to survive, that reflected the high maternal and infant mortality rates in Georgia. Severe social conditions contributed to: chronic depression and intimate partner violence; early sexual activity with sexually transmitted infections and unplanned and/or unwanted pregnancies often with short interpregnancy intervals; poor educational achievement; and unstable housing with crowding and substandard living conditions. The potential impact on infants and children from these factors was well documented in the Adverse Childhood Experiences (ACE) study [36]. Profound negative impacts upon health, social, and economic wellbeing were known to result from childhood trauma [37]. These maternal participants experienced ACE and cascading generational risks that, without further intervention, forecast the lives of themselves and their children.

The dynamics of participants’ decisions were seen in the power struggles to overcome their families’ multi-generational patterns, the struggle for the tax and welfare benefits for the infant’s support, uncontrolled sexual advances against participants, peer pressure, transient housing, the new boyfriend wanting the participant to have his child, and the search for love and belonging by both participants and FOBs [38]. Participants had little power to overcome others’ resistance to their needs to change.

Participants had perpetual toxic lifestyles that families viewed as “normal” [39]. RNCMs’ focus group comments confirmed that families resisted participants’ efforts to change [40]. RNCMs provided a protective relationship by teaching participants to change their responses [41]. RNCMs modeled reflective functioning techniques to encourage participants to think critically and make decisions in terms of potential consequences [28]. Often absent, unemployed, or incarcerated, FOBs had little economic, social, or parental input into the participant’s pregnancy or infant care [23]. The RNCM who asked, “Is that all you want?” cast a vision of higher expectations [42]. Participants looked to the RNCMs for answers and support [43]. RNCMs’ discussion of participants’ conditions and dynamics aligned with investigators’ interpretations.

ECHS staff facilitated participants’ obtaining health insurance during pregnancy and the postpartum period to enable access to medical care. Without continuous health insurance, participants began pregnancy and ended postpartum care with uncontrolled medical risks that were magnified by childbearing [2,44,45]. RNCMs’ focus on facilitating insurance coverage, prenatal care, and transportation to appointments explains the adequate to adequate plus level of care received in spite of the challenges in this rural area.

### 4.1. Strengths of the Study

Critically important information was revealed through in-depth examination of the conditions and dynamics in the lives of participants who did not achieve adequate (≥4) maternal health literacy progression by the end of their initial case management period of service. The study revealed the value of intensive nursing care in the home that contributed to the safe maternal and infant outcomes that occurred.

An important strength of the study was found in the research group process that investigators employed. Investigators stated their reflexivity positions early in the research process. Reflexivity meant a statement of their values, their motivations for participating in the research, and their past personal and professional experiences that would influence how they would interpret data [46]. Team meetings were characterized by synergy in focused discussions. Investigators contributed to the intense review, discussion, and evaluation of the complex set of data available for each participant. From the investigators’ interactions there emerged a worksheet for each participant. Notes and decisions were recorded in real time when the team of investigators reached a conclusion. Every voice was heard. Every voice held equal weight in the production of the worksheets.

Investigators adhered to data and not speculation or interpretation. If the RNCM who had recorded the data included an interpretation, it was regarded as a factual component of the data. Investigators shared their multiple perspectives from personal knowledge based on professional experience in evidence-based nursing practice.

Current technology was one key to the success of the ECHS program. Each RNCM and each participant had a cell phone that enabled them to have unlimited communications. The ECHS program leadership had developed and implemented an electronic health record that permitted recording and retrieving data from each contact. The development and publication of the LSP made possible the structure and measurement of maternal health literacy progression. Combined, these technological advances supported nursing care and enabled investigators to measure the impact of care.

Participants in this study were at very high risk of pregnancy related morbidity or mortality, which was of great concern in public health in the U.S. Close examination using mixed methods research revealed the underlying sociodeterminants of risks, protective factors, and dynamics that impacted maternal health literacy progression of participants and their infants. The work of the RNCMs demonstrated the intensive nursing care necessary to help participants overcome their circumstances.

### 4.2. Limitations of the Study

A small study group with a non-randomized study design cannot yield prescriptive information that can be generalized to a broader population. It can provide insights that others may use to inform future research, services, and program evaluation.

## 5. Conclusions 

### 5.1. Summary

In summary, the study described conditions and dynamics that impacted a group of high-risk, low income, Southern U.S. Healthy Start participants who had not been able to achieve criterion—referenced adequacy in maternal health literacy progression. The ECHS program’s RNCMs provided instruction for mothers and babies, encouraged rational decision-making, supported access to health care with insurance coverage, arranged transportation, and encouraged participants to make needed changes. RNCMs succeeded in helping women and infants survive and improve their life circumstances by reducing risks and addressing medical and social needs even though participants did not achieve the criterion-referenced standard for maternal health literacy.

### 5.2. Investigators’ Recommendations

In order to build health equity among rural, Southern, NHB and NHW families, study results indicated the need to:

(a) Increase support for educational programs that enable participants and FOBs to have greater employment opportunities and greater control over the conditions and dynamics of their lives.
(b) Continue home visitation family care with long-term relationships using evidence-informed, intensive nursing, mental health, and other resources that make care accessible, available, and acceptable.
(c) Advocate for sustainable multi-generational policy and legislative changes that enable access to health care for all with individual accountability.

### 5.3. RNCMs’ Recommendations in Their Own Words

In order to prevent maternal and infant deaths, we need to have:

(a) Mental health counselors who go to the home.
(b) Safe places to house teenage girls who are homeless.
(c) Intensive nursing services for the extremely high-risk women who need a lot more attention, need very high-frequency contact, and just need to talk. Long term contact relationships are important to help them stabilize and sustain positive changes.
(d) Wrap-around services for perinatal/postpartum women—counseling, job training, family planning, check-ups, and baby care—all in one place all in one day.
